# Case-control study of adverse childhood experiences and multiple sclerosis risk and clinical outcomes

**DOI:** 10.1371/journal.pone.0262093

**Published:** 2022-01-13

**Authors:** Mary K. Horton, Shannon McCurdy, Xiaorong Shao, Kalliope Bellesis, Terrence Chinn, Catherine Schaefer, Lisa F. Barcellos

**Affiliations:** 1 Division of Epidemiology and Biostatistics, Genetic Epidemiology and Genomics Laboratory, School of Public Health, University of California, Berkeley, CA, United States of America; 2 Computational Biology Graduate Group, University of California, Berkeley, California, United States of America; 3 California Institute for Quantitative Biosciences, University of California Berkeley, Berkeley, CA, United States of America; 4 Kaiser Permanente Division of Research, Oakland, CA, United States of America; Harvard Medical School, UNITED STATES

## Abstract

**Background:**

Adverse childhood experiences (ACEs) are linked to numerous health conditions but understudied in multiple sclerosis (MS). This study’s objective was to test for the association between ACEs and MS risk and several clinical outcomes.

**Methods:**

We used a sample of adult, non-Hispanic MS cases (n = 1422) and controls (n = 1185) from Northern California. Eighteen ACEs were assessed including parent divorce, parent death, and abuse. Outcomes included MS risk, age of MS onset, Multiple Sclerosis Severity Scale score, and use of a walking aid. Logistic and linear regression estimated odds ratios (ORs) (and beta coefficients) and 95% confidence intervals (CIs) for ACEs operationalized as any/none, counts, individual events, and latent factors/patterns.

**Results:**

Overall, more MS cases experienced ≥1 ACE compared to controls (54.5% and 53.8%, respectively). After adjusting for sex, birthyear, and race, this small difference was attenuated (OR = 1.01, 95% CI: 0.87, 1.18). There were no trends of increasing or decreasing odds of MS across ACE count categories. Consistent associations between individual ACEs between ages 0–10 and 11–20 years and MS risk were not detected. Factor analysis identified five latent ACE factors, but their associations with MS risk were approximately null. Age of MS onset and other clinical outcomes were not associated with ACEs after multiple testing correction.

**Conclusion:**

Despite rich data and multiple approaches to operationalizing ACEs, no consistent and statistically significant effects were observed between ACEs with MS. This highlights the challenges of studying sensitive, retrospective events among adults that occurred decades before data collection.

## Introduction

Adverse Childhood Experiences (ACEs) are potentially traumatic events that occur in childhood and can include physical, emotional, and sexual abuse and/or neglect and household disfunction [1]. They are common in the U.S.—occurring in about 58% of the population—and an important social determinant of health [2]. Childhood represents a particularly vulnerable period when body systems are developing. Excessive activation of stress response systems during this period can impact brain development, immunity, metabolic regulatory systems, and the cardiovascular system [3]. A large body of literature has linked ACEs to physical and mental health conditions in adulthood including heart disease, obesity, type 2 diabetes, cancer, and depression [4].

One particularly relevant downstream effect of excessive activation of stress response systems is dysregulation of the immune system. Numerous studies have shown in experimental and observational settings that psychosocial stressors can cause persistent inflammation and suppression of anti-inflammatory compounds [5–7]. Dysregulation of the immune system can lead to many serious health conditions, including autoimmune conditions such as multiple sclerosis (MS), lupus, and rheumatoid arthritis. The literature regarding the effects of ACEs on autoimmune disorders is limited but suggests an increased numbers of ACEs are associated with increased risk of autoimmune conditions overall and individually [8]. More studies are needed to fully understand this relationship, particularly among individual autoimmune conditions.

MS is one autoimmune condition where more work on this topic is needed. MS is a chronic, inflammatory autoimmune condition of the central nervous system and is the second most common neurological disorder among young adults [9,10]. Diagnosis is common relatively early in adulthood (ages 20 to 40 years) and among women (3:1 female-to-male ratio). Several studies have shown that risk factors (e.g., obesity, concussion, Epstein-Barr virus infection, and vitamin D/sun exposure), particularly during adolescence (ages 11–20 years), are associated with increased MS risk [11,12]. Given the relatively young age of MS diagnosis, support for adolescent exposures being important for MS risk, and the critical involvement of inflammation in MS disease processes, determining whether a role exists for ACEs in MS risk is important. Of the few studies that have examined the association between ACEs and MS risk, their results are inconsistent. Findings from the U.S.-based Nurses’ Health Study, which asked adult participants to quantify “physical or sexual abuse in childhood or adolescence”, suggested MS risk was not significantly associated with abuse [13]. A Danish study found that parent divorce, but not parent or sibling death, was associated with risk of MS [14]. A German study using a 28-item self-report questionnaire of childhood maltreatment found an increased risk of MS among domains of physical abuse, sexual abuse, emotional neglect, and severe abuse [15]. Inconsistencies in these findings are likely due to differences in specific ACEs and how they were quantified, as well as differing cultural and social contexts in each population, underscoring the challenges of this important work and need for further investigation.

There is even less knowledge about whether ACEs affect clinical outcomes of MS which may be influenced by early life stress and inflammation. The largest study, to date, to investigate this association utilized 217 MS cases and determined that physical abuse, emotional neglect, and severe abuse were associated with higher relapse rates but not age of onset or other indicators of physical or cognitive outcomes [15]. The only other (smaller) study to investigate this found that more ACEs were associated with younger age of MS onset and worse reading cognition [16]. Understanding the relationship between ACEs and MS risk and clinical outcomes may strengthen the argument for childhood screening of ACEs and interventions that prevent or modify the effects of ACEs and improve our understanding of MS etiology.

Our approach to studying ACEs was to interrogate how they might affect MS risk and clinical outcomes using multiple methodologies. For our study, ACEs included death of a parent or sibling, victim of a violent crime, loss of a home, and significant physical or verbal abuse or neglect, among others. It is common to analyze ACEs as individual events or summarized into any/none or count variables; however, these have several limitations. It is possible that individual ACEs (such as parent divorce shown by Riise et.al) may have different effects on MS risk, but ACEs (and social exposures more broadly) often co-occur and are not necessarily independent [17]. This limits the interpretability of assessing single events that are highly inter-related. In addition, single events may be rare and limit our power to examine associations with MS in all but very large studies. Quantifying ACEs as any/none may be meaningful if the hypothesis is that any adverse event impacts health. However, this dichotomy fails to consider the relative importance of different types of ACEs with varying impacts on chronic stress or behaviors and thus MS. The use of counts assumes the cumulative burden of ACEs affects health, rather than particular type, combination, or chronicity. These limitations highlight the challenges in studying ACEs and the need to consider them in multiple ways in order to understand their complex, nuanced relationships with health outcomes, particularly MS.

The aim of the current study was to estimate the association between ACEs and MS risk and several clinical outcomes, including age of onset, use of a walking aid, and Multiple Sclerosis Severity Scale score, in a case-control sample of 2607 adults in Northern California using multiple approaches including quantifying ACEs as individual events, any/none, and counts. We also included a factor analysis to evaluate variance of ACEs in order to identify “latent factors”, which are weighted linear combinations of variables, that represent patterns of ACEs that tend to co-occur. Collectively, this approach may help identify how ACEs are associated with MS.

## Methods

### Study population

Data were from the Kaiser Permanente Northern California (KPNC) MS Research Program which recruited non-Hispanic MS cases and controls from the KPNC Health Plan between 2006 and 2014. This membership includes over four million people, representing 25–30% of the 22-county service area population in Northern California. The broad goal of this study was to assess risk factors for MS across hundreds of genetic and environmental exposures. To achieve sufficient power for genetic analyses, study inclusion was limited to the largest subgroup of KPNC members which were largely non-Hispanic whites. Recruitment details are explained elsewhere [18]. Briefly, eligible cases were diagnosed with MS by a neurologist (*International Classification of Diseases*, *Ninth Revision*, code 340.x), aged 18–69 years old, and a KPNC member at initial contact. For our analyses, cases were excluded if age of onset occurred before age 21 years to minimize the potential for reverse causality or MS onset occurring before ACEs (assessed up to age 20). Age of onset was determined by review of electronic health records and comprehensive interview data. Controls were KPNC members without a MS diagnosis or related condition (optic neuritis, transverse myelitis, or demyelinating disease) and were matched to cases by sex, age, and zip code. A total of 2607 participants (1422 cases and 1185 controls) were available for analyses.

Study protocols for participants were approved by the Institutional Review Boards of KPNC and the University of California, Berkeley. Written informed consent was obtained from all study participants.

### Adverse Childhood Experiences (ACEs)

Participants were administered a comprehensive computer-assisted telephone interview (CATI) including hundreds of self-reported demographic, clinical, environmental, and lifestyle questions, as described elsewhere [19]. The CATI included nine ACE questions modified from Coddington’s Life Event Record [20] (Table 1). Not all questions were included to reduce the length of the extensive CATI. Events included broadly overlap with the original Centers for Disease Control and Prevention-Kaiser ACE Study [1], but there are several differences. Our study does not ask about sexual abuse, household substance abuse, or incarcerated household members. It also combines physical and verbal abuse and adds questions about parent/sibling death and foster care/adoption. Participants indicated yes/no as to whether they experienced any of the events in either of two age periods: 0–10 and 11–20 years old (a total of 18 ACEs). These two time periods were chosen because studies have shown that relationships between several risk factors and MS differ in adolescence and childhood [11,12].

**Table 1:** Definition and baseline prevalence of adverse childhood experiences reported by adult multiple sclerosis (MS) cases and controls in the Kaiser Permanente Northern California MS Research Program, 2006–2014 (n = 2607).

	Adverse Childhood Experience	Total		MS Cases		Controls	
		No.	%	No.	%	No.	%
Remembering back to your early childhood through the age of 10, did you experience any of the following list of events?							
1	Death of parent or sibling	97	3.7	44	3.1	53	4.5
2	Divorce of parents	275	10.5	145	10.2	130	11.0
3	Remarriage of parents	183	7.0	95	6.7	88	7.4
4	Placed in foster care or adoption	57	2.2	34	2.4	23	1.9
5	Went to live with other family members	149	5.7	86	6.1	63	5.3
6	Serious (life-threatening) illness of parent or sibling (including psychiatric illness or substance abuse problem)	312	12.0	170	12.0	142	12.0
7	You experienced significant physical or verbal abuse or neglect	315	12.1	143	10.1	172	14.5
8	Your family lost their home or had to move	196	7.5	85	6.0	111	9.4
9	You were the victim of a violent crime	58	2.2	32	2.3	26	2.2
Remembering back to when you were a teenager, between 11 until you turned 20 years old, did you experience any of the following list of events?							
10	Death of parent or sibling	156	6.0	93	6.5	63	5.3
12	Divorce of parents	266	10.2	140	9.9	126	10.6
13	Remarriage of parents	232	8.9	119	8.4	113	9.5
13	Placed in foster care or late adoption	44	1.7	31	2.2	13	1.1
14	Went to live with other family members	205	7.9	117	8.2	88	7.4
15	Serious (life-threatening) illness of parent or sibling (including psychiatric illness or substance abuse problem)	367	14.1	203	14.3	164	13.8
16	You experienced significant physical or verbal abuse or neglect	389	14.9	200	14.1	189	15.9
17	Your family lost their home or had to move	184	7.1	98	6.9	86	7.3
18	You were the victim of a violent crime	130	5.0	71	5.0	59	5.0

Abbreviations: MS, multiple sclerosis.

### MS clinical outcomes

As part of the CATI, MS cases were asked the year of their first MS symptom (i.e., “onset”), the type of MS they currently have (relapsing-remitting, secondary progressive, primary progressive, or relapsing-progressive), and an indication of their walking ability in the past four weeks. For each MS case, we calculated the Multiple Sclerosis Severity Scale (MSSS), which is an indicator of disease severity that uses the Expanded Disability Severity Scale and disease duration (time from onset to EDSS) [21]. We also created an indicator of whether a case had severe or mild MS based on MSSS scores (≥7.5 was severe and <3 was mild).

### Covariates

Demographic and clinical data collected from the CATI and considered confounders included sex, birth year, race, and years since MS onset. Race was categorized as white or non-white, noting 98.5% of non-whites identified as African American. Additional confounders considered in sensitivity analyses (see below) included education-level (bachelor’s degree or not), parent’s homeowner status when participant was 10 years old (rent vs own/other), and family history of MS (parent or sibling). These were not included in primary/secondary analyses to preserve statistical power and prevent over-stratification of models with already low frequency substrata (including rare ACEs, men, and non-whites).

### Statistical analysis

Factor analysis was conducted among all participants to determine the latent factor structure of 18 total ACEs (nine ACEs at two time points). A tetrachoric correlation matrix, appropriate for binary data, was constructed. Zero-count cells were corrected by adding 0.1. Factors were extracted using maximum likelihood estimation in the *polycor* package and *factanal* in R Version 3.5 [22]. VARIMAX (orthogonal) rotation was used to increase interpretability of factors. Number of factors to extract was based on optimal coordinates and reduced if any factor loading was ≥1.0 [23]. Factor scores were calculated and standardized to a mean of zero and standard deviation of one [24].

Primary analyses tested the association between ACEs and MS risk using logistic regression to estimate odds ratios (ORs) and 95% confidence intervals (CIs). ACEs were expressed as: 1) any/none at each time period and overall, 2) 0, 1, 2, 3, or 4 or more at each time period and overall, 3) individually at each time period and overall, and 4) continuously for each factor score. The associations between individual ACEs and MS risk were only estimated for ACEs that occurred in at least 5% of the sample in order to achieve sufficient statistical power. To improve interpretability of ORs from models using continuous factor scores (where a 1-unit increase in respective factor score would represent nearly the entire range of values), beta coefficients and their standard errors were divided by ten. All models adjusted for sex, birthyear, and race. Multiple testing corrected false discovery rate (FDR) *q* values are presented for primary analyses [25]; they account for all primary models assessing MS risk simultaneously. All analyses used R Version 3.5 [22].

Secondary analyses investigated the association between ACEs and clinically relevant MS outcomes including MSSS, age of onset, progressive MS subtype, and current walking ability. We also included a sub-analysis comparing ACEs among individuals with mild and severe MS. For MSSS and age of onset outcomes, linear regression models were used to estimate beta coefficients and 95% CIs. Both models adjusted for sex and race. MSSS models additionally adjusted for birthyear. Age of onset was approximately normally distributed while MSSS was slightly right-skewed. We also conducted a sub-analysis utilized individuals only at the extreme ends of the MSSS scale (n = 818) where the outcome was severe or mild (reference) illness. For MS subtype, type of MS was categorized as relapsing (relapsing remitting or secondary progressive) (reference) or progressive (primary progressive or relapsing progressive). For current walking ability, individuals were classified according to whether they did or did not (reference) regularly use a walking aid (such as cane, walker, or wheelchair). For all binary outcomes, ORs and 95% CIs were estimated using logistic regression and adjusted for birthyear, sex, and race. Walking ability models additionally adjusted for years since MS onset. For all MS outcome models, ACEs were considered the independent variable and expressed as count categories (0, 1, 2, 3, or 4 or more) over the entire exposure period (0 through 20 years of age). Additional ACE classifications were not included to minimize the impact of multiple testing corrections on a reduced sample size (1422 MS cases). Results from secondary analyses were corrected for FDR and account for all secondary clinical outcome tests.

### Sensitivity analyses

To evaluate whether socioeconomic factors independent of race might confound the observed primary associations between ACEs and MS risk, we included two additional logistic regression models which adjust for covariates in the original models plus 1) participant’s educational level or 2) parent’s homeowner status when participants were 10 years old and family history of MS. Family history was considered a potential confounder because the risk of MS is ~seven times higher among those who have a first degree relative with MS [26] and it may be a cause of parent or sibling illness or death (an ACE in our assessment).

## Results

Baseline characteristics were described in Table 2. Among MS patients, 79.0% identified as female (81.5% for controls). The average years since MS onset was 17.1 (sd = 11.8), and the majority of MS cases had mild illness (MSSS <3) (47.6%). Cases had higher frequency of family history of MS (6.8%) compared to controls (1.6%), as expected. When participants were 10 years old, fewer parents of MS cases owned a home compared to controls (78.0% and 81.9%, respectively), as previously reported [19].

**Table 2:** Baseline characteristics among multiple sclerosis (MS) cases and controls in the Kaiser Permanente Northern California MS Research Program, 2006–2014 (n = 2607).

Characteristic	MS Cases (n = 1422)		Controls (n = 1185)	
	No.	%	No.	%
Birth year (mean, sd)	1958 (8.8)		1958 (8.9)	
Sex (female)	1,124	79.0	966	81.5
Parent Homeowner Status at 10 years old				
Own	1109	78.0	970	81.9
Rent/Other Arrangement	298	21.0	210	17.7
Not available	15	1.0	5	0.4
Race^a^				
White	1288	90.6	1114	94.0
African American	134	9.4	72	6.0
American Indian or Alaskan native	3	0.2	0	0.0
Family history of MS^b^ (yes)	97	6.8	19	1.6
ACEs, count (mean, sd)	1.3 (1.8)		1.4 (1.9)	
ACEs, count categories				
0	647	45.5	548	46.2
1	293	20.6	189	15.9
2	224	15.8	188	15.9
3	97	6.8	103	8.7
4 or more	161	11.3	157	13.2
Years since MS onset (mean, sd)	17.1 (11.8)			
MSSS (mean, sd)	3.8 (2.5)			
MSSS <3	677	47.6		
MSSS ≥7.5	141	9.9		
MS subtype				
Relapsing remitting	938	66.0		
Primary progressive	113	7.9		
Secondary progressive	221	15.5		
Relapsing progressive	51	3.6		
Unknown	99	7.0		

Abbreviations: ACEs, adverse childhood experiences; MS, multiple sclerosis; MSSS, Multiple Sclerosis Severity Score.

^a^Two individuals reported American Indian/Alaskan Native and white race, one reported American Indian/Alaskan Native and African American race, and one reported African American and white race.

^b^Defined as having a parent or sibling with MS.

The proportion of participants who experienced ≥1 ACE was higher among cases (54.5%) compared to controls (53.8%) (Table 2). Among the entire sample, the most common ACE during ages 0–10 years was significant physical abuse/neglect (12.1%); it was also the most common ACE during ages 11–20 year (14.9%) (Table 1). The distribution of individual ACEs was similar among cases and controls although fewer cases reported significant physical abuse/neglect or home loss during ages 0–10 years.

Overall, individuals who reported at least one ACE between ages 0–20 years did not have a significantly higher odds of MS compared to individuals who experienced none (OR = 1.01, 95% CI: 0.87, 1.18) (Table 3). A similar non-significant effect was also observed for each age category separately. When ACE counts were categorized into 0, 1, 2, 3, or 4 or more, none of the categories were significantly associated with MS and there were no consistent trends where increased ACEs increased or decreased odds of MS. No individual ACEs were significantly associated with MS at an FDR *q*<0.05 except abuse (OR = 0.66, 95% CI: 0.52, 0.84) and home loss (OR = 0.61, 95% CI: 0.45, 0.82) between ages 0–10 years. These effect sizes were attenuated and not statistically significant at ages 11–20 years (OR_abuse_ = 0.87, 95% CI: 0.70, 1.08 and OR_home loss_ = 0.96, 95% CI: 0.71, 1.30). For secondary analyses pertaining to ACEs and clinical outcomes of MS, no associations were significant at FDR *q*<0.05 (Table 4). Before adjusting for multiple testing comparisons, two associations were significant at *p* <0.05. These included a two year younger age of onset, on average, for MS cases who experienced at least four ACEs compared to those who experienced no ACEs (β = -1.99, 95% CI: -3.62, -0.37, *p* = 0.02), and a higher odds of needing to regularly use a walking aid among MS cases who experienced at least four ACEs compared to MS cases who experienced no ACEs (OR = 1.52, 95% CI: 1.03, 2.24, *p* = 0.03).

**Table 3:** Results from multivariable logistic regression models of the effect of adverse childhood experiences (ACEs) during two age periods on odds of multiple sclerosis.

	Overall			Ages 0–10 years			Ages 11–20 years		
Model	OR	95% CI	FDR *q*	OR	95% CI	FDR *q*	OR	95% CI	FDR *q*
At least one ACE (ref = none)	1.01	0.87, 1.18	0.96	0.86	0.73, 1.01	0.34	1.03	0.88, 1.21	0.95
Count category									
0 ACEs (ref)	1.00	-	-	1.00	-	-	1.00	-	-
1 ACE	1.29	1.04, 1.61	0.17	1.02	0.83, 1.24	0.96	1.12	0.93, 1.35	0.59
2 ACEs	0.99	0.79, 1.24	0.97	0.70	0.54, 0.92	0.12	0.87	0.67, 1.13	0.63
3 ACEs	0.78	0.57, 1.05	0.38	0.59	0.39, 0.89	0.12	0.96	0.67, 1.38	0.96
4 or more ACEs	0.86	0.67, 1.10	0.59	0.87	0.55, 1.41	0.83	1.05	0.68, 1.63	0.96
Individual events									
Parent/sibling death	0.95	0.73, 1.25	0.95	-	-	-	1.22	0.88, 1.71	0.59
Parent divorce	0.86	0.70, 1.05	0.48	0.88	0.68, 1.14	0.63	0.91	0.71, 1.18	0.78
Parent remarries	0.86	0.69, 1.08	0.59	0.88	0.65, 1.19	0.69	0.87	0.66, 1.14	0.63
Live elsewhere	1.13	0.89, 1.43	0.63	1.12	0.80, 1.57	0.78	1.10	0.82, 1.47	0.78
Parent/sibling illness	1.02	0.84, 1.23	0.96	1.02	0.80, 1.29	0.96	1.04	0.84, 1.30	0.95
Abuse	0.82	0.67, 1.01	0.31	0.66	0.52, 0.84	0.02	0.87	0.70, 1.08	0.59
Home lost	0.79	0.62, 1.01	0.31	0.61	0.45, 0.82	0.02	0.96	0.71, 1.30	0.96
Violent crime	1.00	0.74, 1.38	0.98	-	-	-	0.98	0.69, 1.40	0.96
Latent variables									
Factor 1	0.98	0.95, 1.02	0.76	-	-	-	-	-	-
Factor 2	0.99	0.96, 1.01	0.63	-	-	-	-	-	-
Factor 3	0.99	0.97, 1.01	0.63	-	-	-	-	-	-
Factor 4	1.07	1.01, 1.14	0.20	-	-	-	-	-	-
Factor 5	0.97	0.92, 1.00	0.36	-	-	-	-	-	-

All models adjusted for birthyear, sex, and race. ORs for individual ACEs that did not occur in at least 5% of samples were not estimated. Beta coefficients, standard errors, and their respective ORs and 95% CIs were scaled to 0.1-unit increases for factor scores.

Abbreviations: ACEs, adverse childhood experiences; CI, confidence interval; FDR, false discovery rate; OR, odds ratio.

**Table 4:** Results from multivariable regression models of the effect of adverse childhood experiences (ACEs) during ages 0–20 years on clinical outcomes of multiple sclerosis.

	1 vs 0 ACEs			2 vs 0 ACEs			3 vs 0 ACEs			4 or more vs 0 ACEs			
Outcome	Beta/OR	95% CI	FDR *q*	Beta/OR	95% CI	FDR *q*	Beta/OR	95% CI	FDR *q*	Beta/OR	95% CI	FDR *q*	
MSSS^a^	0.31	-0.03, 0.65	0.27	0.34	-0.04, 0.71	0.27	0.04	-0.49, 0.56	0.91	0.30	-0.13, 0.72		0.44
Age at onset^b^	0.28	-1.03, 1.58	0.90	-0.43	-1.86, 1.00	0.90	-1.03	-3.04, 0.97	0.62	-1.99	-3.62, -0.37		0.27
Progressive course^a^	1.12	0.72, 1.72	0.90	0.96	0.59, 1.53	0.91	0.96	0.46, 1.85	0.91	0.87	0.46, 1.53		0.90
Use of walking aid^c^	1.29	0.94, 1.76	0.33	1.23	0.87, 1.74	0.53	0.86	0.51, 1.42	0.90	1.52	1.03, 2.24		0.27
Severe illness	1.57	0.97, 2.52	0.27	1.59	0.94, 2.66	0.27	1.07	0.46, 2.23	0.91	0.91	0.43, 1.79		0.91

Beta coefficients were presented for continuous outcomes (MSSS, age off onset) while ORs were presented for binary outcomes.

^a^Models adjusted for birthyear, sex, and race.

^b^Model adjusted for sex and race.

^c^Models adjusted for birthyear, sex, race, and years since MS onset.

Abbreviations: ACEs, adverse childhood experiences; CI, confidence interval; FDR, false discovery rate; MSSS, Multiple Sclerosis Severity Score; OR, odds ratio; *q*, *q-value*.

Optimal coordinates analysis identified five factors of co-occurring ACEs which explained 57.0% of the variance in 18 reported ACEs (S1 Table). For each factor, the following ACEs contributed the largest loadings: lost home or moved ages 0–10 and 11–20 years (Factor 1), parent divorce and parent remarriage ages 0–10 (Factor 2), physical or verbal abuse or neglect ages 0–10 and 11–20 years (Factor 3), placed in foster care and parents divorced ages 11–20 years (Factor 4), and parent or sibling death ages 0–10 years (Factor 5). Logistic regression using continuous factor scores did not yield statistically significant results (Table 3). For all factors, a 0.1-unit increase in factor score had very small or null association with MS risk (e.g., Factor 1 OR = 0.98, 95% CI: 0.95, 1.02).

Sensitivity analyses for MS risk models yielded ORs and 95% CIs that did not substantially change when models additionally controlled for participant’s educational attainment, parent’s homeowner status, or family history of MS (S2 and S3 Tables).

## Discussion

ACEs are associated with numerous adult health conditions [4], but the relationship between ACEs and MS has remained elusive. Understanding this relationship may be particularly relevant because one hypothesized biological mechanism linking ACEs and general poor adult health is inflammation [27], a key cause of neuronal damage in MS. Despite rich data and multiple approaches for operationalizing ACEs in the current study, no consistent and statistically significant effects were observed between ACEs with MS risk and clinical outcomes after correcting for multiple testing comparisons. This highlights the challenges of studying sensitive, retrospective events among adults that occurred decades before data collection. It also underscores the need for ACE assessments early in the MS disease course to overcome some of these challenges.

Our primary findings, which do not support the role of ACEs in risk of MS, both agree with and contradict past studies of MS and autoimmune disorders. Results from a large cohort study of U.S. nurses did not identify associations between MS and stressful life events, including physical and/or sexual abuse during childhood or adolescence [13]. Corresponding odds ratios ranged from 0.72 to 1.30 but were not statistically significant, which may be due to the small number of MS cases identified from the large cohort (n = 369). These findings align with the magnitude and insignificant nature of the current findings. Similar to our results, a Danish study (the largest study to date) found that risk of MS was not associated with parent death (OR = 1.04, 95% CI: 0.90, 1.21) or sibling death (OR = 1.04, 95% CI: 0.81, 1.32) [14]. However, this study did observe that parent divorce, specifically, was associated with increased risk of MS (OR = 1.13, 95% CI: 1.04, 1.23), which is not consistent with our results. Their results are likely highly accurate given that Danish registries capture all family relations and marital statuses for all Danish residents and capture all MS diagnoses since 1956. However, social structures, levels of inequities, and the demographic make-up of Denmark and the U.S. are very different, so these adverse events might not be expected to have the same effects in both countries. Our findings pertaining to physical abuse (and home loss) demonstrated a significant protective effect during childhood, but there is no reason to believe that physical abuse or home loss, but not other ACEs, would prevent MS. In fact, previous research contradicts this finding which identified an increased risk of MS among those who have experienced severe abuse (OR = 1.7) and null associations between physical abuse or neglect and MS risk [15]. Similarly, latent factors 1 or 3 were not associated with MS risk despite being the factors for which childhood abuse and home loss contributed the most.

Among other autoimmune conditions, increasing number of ACEs have been associated with first hospitalization of any autoimmune disease as well as rheumatic, Th1-type and Th2-type immunopathologies, and Systemic Lupus Erythematosus (SLE) [8,28]. In particular, physical and emotional abuse have been shown to be associated with over two times the risk of SLE [8]. These were not found to be associated in our study. The differing results may be a result of different associations between ACEs and specific autoimmune conditions or insufficient statistical power, measurement error, or selection bias within our study or others.

Our findings that a younger age of onset and regular use of a walking aid were more common among MS cases that had at least four ACEs were not significant after correcting for multiple testing comparisons. Current research on this topic is very limited, with only two small studies reporting their findings. Of these, age of onset was found to be inversely correlated with ACEs (r = −0.30, p = 0.04) [16] or not associated with ACEs [15]. In another autoimmune condition, SLE, higher ACE levels and ACE domains were associated with worse patient-reported disease activity, depression, and health status [29]. It is important to note that our analysis did not have available comprehensive clinical outcomes data; therefore, only several features were assessed. Additional analyses considering relapse rate, neuroimaging measures, symptom burden, fatigue, pain, cognitive impairment, health-related quality of life, and psychological impacts might reveal meaningful associations with ACEs and should be conducted in the future. Our findings should be explored further in a larger sample size to improve statistical power to identify whether a true relationship exists between clinical features of MS and ACEs.

A major challenge that may have contributed to inconsistencies between our results and other studies, as well as our generally null observed effects, is information bias. Particularly, retrospectively asking adults about ACEs that occurred decades in the past that are sensitive by nature and may be misremembered or repressed from memory could have led to underreporting. Comparing the frequency of several of our study’s ACEs to those in the Behavioral Risk Factor Surveillance System (BRFSS) (derived from the Kaiser-CDC ACEs study) provides evidence of this underreporting. For example, 28% and 34% of individuals in the BRFSS had their parents’ divorce/separate and experienced emotional abuse while 19% and 18% experienced these ACEs in our sample, respectively [30]. Recall of sensitive events may have been under-reported, specifically, among cognitively impaired MS patients. However, this is not consistent with knowledge that cognitive MS symptoms do not commonly affect recall of memories from the distant past but rather lead to trouble with recall due to deficits in ability to store new knowledge for future recall [31,32]. Alternatively, MS cases may have interpreted questions regarding home loss or abuse more conservatively than controls, not willing to report the event unless they considered it an extreme circumstance. This is unlikely given “recall bias” which often, but not always, leads to more accurate recall of particular events/exposures among case groups than control groups.

In addition to this potential retrospective reporting bias, there are several limitations that should be considered. First, the events utilized in this dataset are not, together, part of a standardized ACE index. Compared to the BRFSS, our events similarly included parent divorce/separation, but did not include substance use, parent incarceration, or sexual abuse. Exclusion of these sensitive, important topics may have contributed to observed null findings. This is particularly relevant given that household substance abuse is one of the more common ACEs in the BRFSS (26.8% reported experiencing this) [2]. Combining physical and verbal abuse into a single category may also have underestimated the impact of ACEs in our sample. We did, however, include important events not part of the BRFSS survey including parent death and life-threatening illness of parent or sibling. Second, using ACEs is an imperfect way of measuring childhood adversity. Individual events tend to be interrelated and the social environment and factors that may influence it are complex and challenging to disentangle. To improve upon individual ACE analyses (which also may suffer from reduced statistical power due to rarity of certain events), we utilized factor analysis to create unobserved “latent” variables to capture the relatedness of ACEs. The observed associations between each latent variable and MS risk were approximately null, but the extent to which these factors might represent true unobserved continuous variables remains unknown. These five factors captured a relatively small amount of variation in ACEs (57%), which also limits the effectiveness of estimating their associations with MS. Last, low income and African American individuals disproportionately experience a number of adverse experiences [30,33]. This demographic is under-represented in the current sample which may lead to limited generalizations of findings to more diverse populations or selection bias. It may also have led to the observed null findings given African Americans tend to have worse MS clinical outcomes compared to Whites [34]. Future studies should further explore relationships between ACEs and MS among African Americans, Hispanics, Asians, and other non-White populations. This work is currently underway. Future studies should also investigate the nuanced synergistic and/or cumulative relationships between ACEs, socioeconomic position, and MS. For example, the effect of ACEs on MS may be stronger among individuals whose parents rented rather than owned a home (indicator of socioeconomic position and associated with MS) or among those who also experienced stressful events as adults later in the lifespan [19].

## Conclusions

Findings from the current study did not support an association between ACEs and development of MS or clinical feature of MS. While we cannot exclude the potential role of ACEs on MS, our results highlight how poor recall or even recall bias for reporting sensitive events in the past may be particularly challenging to overcome in the context of MS. Future studies should consider alternative tools for assessing ACEs and childhood trauma, such as biomarkers of stress, and/or obtain ACE information from MS patients as close to diagnosis as possible to reduce the number of years between exposure and outcome.

## Supporting information

S1 Table Factor loadings for a 5-factor model based on adverse childhood experiences data from the Kaiser Permanente Northern California Multiple Sclerosis Research Program cases and controls, 2006–2014 (n = 2,607). (PDF)

S2 Table Sensitivity analysis of multivariable logistic regression models of the effect of adverse childhood experiences (ACEs) during two age periods on odds of multiple sclerosis accounting for educational attainment. (PDF)

S3 Table Sensitivity analysis of multivariable logistic regression models of the effect of adverse childhood experiences (ACEs) during two age periods on odds of multiple sclerosis (MS) accounting for parent homeowner status and family history of MS. (PDF)
