# Supplementary material for: Case-control study of adverse childhood experiences and multiple sclerosis risk and clinical outcomes
Source: PLoS One. 2022 Jan 13;17(1):e0262093. doi: 10.1371/journal.pone.0262093 (PMC8757911; doi:10.1371/journal.pone.0262093)
Supplement: S1 Table — (PDF) [file pone.0262093.s001.pdf]

S1 Table. Factor loadings for a 5-factor model based on adverse childhood experiences data from the Kaiser Permanente Northern California Multiple Sclerosis Research Program cases and controls, 2006-2014 (n=2,607).

	Item	Factor 1	Factor 2	Factor 3	Factor 4	Factor 5
Ages 0-10 years	Death of parent or sibling	0.16	0.11	0.04	0.02	0.98
	Divorce of parents	0.15	0.96	0.17	0.13	-0.12
	Remarriage of parents	0.02	0.85	0.12	0.10	0.28
	You were placed in foster care (or late adoption)	0.20	0.14	0.04	0.72	-0.08
	Went to live with other family members	0.46	0.28	0.15	0.30	0.19
	Serious (life-threatening) illness of parent or sibling (including psychiatric illness or substance abuse problem)	0.46	0.05	0.12	0.00	0.35
	You experienced significant physical or verbal abuse or neglect	0.29	0.21	0.79	0.18	0.08
	Your family lost their home or had to move	0.60	0.33	0.20	0.11	0.06
	You were the victim of a violent crime	0.49	0.18	0.26	0.17	0.07
Ages 11-20 years	Death of parent or sibling	0.29	0.00	0.01	-0.03	0.07
	Divorce of parents	0.43	0.24	0.13	-0.52	0.01
	Remarriage of parents	0.42	0.57	0.09	-0.13	0.07
	You were placed in foster care (or late adoption)	-0.14	0.09	0.18	0.90	0.12
	Went to live with other family members	0.31	0.27	0.19	0.19	0.01
	Serious (life-threatening) illness of parent or sibling (including psychiatric illness or substance abuse problem)	0.45	0.02	0.16	-0.12	0.04
	You experienced significant physical or verbal abuse or neglect	0.26	0.11	0.95	0.03	0.08
	Your family lost their home or had to move	0.63	0.14	0.19	0.02	-0.03
	You were the victim of a violent crime	0.45	0.16	0.41	-0.02	-0.10
	Proportion of variance explained	0.15	0.14	0.11	0.10	0.07
	Cumulative variance explained	0.15	0.29	0.40	0.50	0.57
