# Supplementary material for: Case-control study of adverse childhood experiences and multiple sclerosis risk and clinical outcomes
Source: PLoS One. 2022 Jan 13;17(1):e0262093. doi: 10.1371/journal.pone.0262093 (PMC8757911; doi:10.1371/journal.pone.0262093)
Supplement: S2 Table — (PDF) [file pone.0262093.s002.pdf]

S2 Table. Sensitivity analysis of multivariable logistic regression models of the effect of adverse childhood experiences (ACEs) during two age periods on odds of multiple sclerosis accounting for educational attainment.

	Overall		Ages 0-10 years		Ages 11-20 years	
Model	OR	95% CI	OR	95% CI	OR	95% CI
At least one ACE (ref=none)	0.98	0.84, 1.15	0.83	0.71, 0.98	1.00	0.86, 1.18
Count category						
0 ACEs (ref)	1.00	-	1.00	-	1.00	-
1 ACE	1.26	1.02, 1.57	0.99	0.81, 1.21	1.11	0.92, 1.33
2 ACEs	0.96	0.77, 1.21	0.68	0.52, 0.90	0.83	0.64, 1.08
3 ACEs	0.74	0.55, 1.01	0.56	0.47, 0.85	0.92	0.64, 1.32
4 or more ACEs	0.81	0.63, 1.04	0.82	0.50, 1.32	0.99	0.64, 1.54
Individual events						
Parent/sibling death	0.92	0.70, 1.21	-	-	1.18	0.85, 1.65
Parent divorce	0.83	0.68, 1.01	0.85	0.66, 1.09	0.88	0.68, 1.14
Parent remarries	0.84	0.67, 1.05	0.83	0.61, 1.13	0.86	0.65, 1.13
Live elsewhere	1.07	0.84, 1.37	1.07	0.76, 1.50	1.04	0.78, 1.40
Parent/sibling illness	1.02	0.84, 1.24	1.03	0.81, 1.31	1.04	0.83, 1.31
Abuse	0.79	0.65, 0.97	0.64	0.50, 0.81	0.85	0.68, 1.05
Home lost	0.77	0.60, 0.99	0.60	0.44, 0.80	0.93	0.68, 1.26
Violent crime	0.99	0.72, 1.36	-	-	0.97	0.68, 1.40
Latent variables			-	-	-	-
Factor 1	0.98	0.94, 1.02				
Factor 2	0.99	0.96, 1.01	-	-	-	-
Factor 3	0.99	0.97, 1.01	-	-	-	-
Factor 4	1.07	1.00, 1.13	-	-	-	-
Factor 5	0.96	0.93, 1.00	-	-	-	-

Total number of participants without missing covariate data is 2,603. All models adjusted for year of birth, sex, race (white or non-white), and educational attainment. ORs for individual ACEs that did not occur in at least 5% of samples were not estimated. Beta coefficients, standard errors, and their respective ORs and 95% CIs were scaled to 0.1-unit increases for factor scores.

Abbreviations: ACEs, adverse childhood experiences; CI, confidence interval; OR, odds ratio
